# In Situ Water Quality Monitoring Using an Optical Multiparameter Sensor Probe

**DOI:** 10.3390/s23239545

**Published:** 2023-11-30

**Authors:** Tobias Goblirsch, Thomas Mayer, Stefanie Penzel, Mathias Rudolph, Helko Borsdorf

**Affiliations:** 1 UFZ Helmholtz Centre for Environmental Research, Department Monitoring and Exploration Technologies, Permoserstraße 15, 04318 Leipzig, Germany; thomas.mayer@ufz.de (T.M.); helko.borsdorf@ufz.de (H.B.); 2 Faculty of Engineering, Leipzig University of Applied Sciences (HTWK Leipzig), Karl-Liebknecht-Straße 134, 04277 Leipzig, Germany; stefanie.penzel@htwk-leipzig.de (S.P.); mathias.rudolph@htwk-leipzig.de (M.R.)

**Keywords:** UV/Vis spectroscopy, fluorescence spectroscopy, in situ water monitoring, model–view–controller architecture, submersible sensor probe

## Abstract

Optical methods such as ultraviolet/visible (UV/Vis) and fluorescence spectroscopy are well-established analytical techniques for in situ water quality monitoring. A broad range of bio-logical and chemical contaminants in different concentration ranges can be detected using these methods. The availability of results in real time allows a quick response to water quality changes. The measuring devices are configured as portable multi-parameter probes. However, their specification and data processing typically cannot be changed by users, or only with difficulties. Therefore, we developed a submersible sensor probe, which combines UV/Vis and fluorescence spectroscopy together with a flexible data processing platform. Due to its modular design in the hardware and software, the sensing system can be modified to the specific application. The dimension of the waterproof enclosure with a diameter of 100 mm permits also its application in groundwater monitoring wells. As a light source for fluorescence spectroscopy, we constructed an LED array that can be equipped with four different LEDs. A miniaturized deuterium–tungsten light source (200–1100 nm) was used for UV/Vis spectroscopy. A miniaturized spectrometer with a spectral range between 225 and 1000 nm permits the detection of complete spectra for both methods.

## 1. Introduction

The application of in situ sensors permits the detection of contaminants in aquatic ecosystems with a high spatial and temporal resolution. This high data density enables the precise localization of contamination or anomalies and their rapid detection [1]. Time series analyses can contribute to the monitoring of inflows to waterworks, for example. Direct measurement in the field avoids a number of measuring errors such as undesirable transfer of volatile compounds into the gas phase, adsorption and reactions leading to non-quantifiable loss of the original substances [2]. Different water quality monitoring technologies for the in situ and real-time detection of biological and chemical contaminants are available today [3,4]. UV/Vis and fluorescence spectroscopy have proven to be versatile and reliable methods for the in situ monitoring of aquatic systems [5,6]. Both methods can detect a variety of substances or sum parameters at different sensitivities. UV/Vis sensors are mainly applied for the monitoring of nitrate [7,8] or dissolved organic carbon (DOC) and some other parameters [9], while fluorescence spectroscopy permits the sensitive detection of microbial contamination [10], cyanobacteria [11], chlorophyll [12] and a few sum parameters such as dissolved organic matter (DOM) [13]. A more detailed overview of the most common applications of both methods and the experimental parameters used are summarized in Table 1. Turbidity indicates the presence of suspended particulate matter and can be detected using UV/Vis spectroscopy at high concentrations (40–4000 FAU; Formazine Attenuation Units), while scattered light measurements permit its more sensitive determination (<0.05–400 FNU; Formazine Nephelometric Units).

Various technical developments are described in the literature with the aim of miniaturizing the sensors and measuring different parameters simultaneously [14]. A number of in situ sensors were developed in scientific projects for special applications such as turbidity sensors [15], portable fluorescence sensors [16], low-cost and smart chlorophyll-A sensors [17], fluorescence spectrometers for DOM [18], fecal contamination sensors [19] and multi-platform sensors [20].

**Table 1:** Water quality parameters measured using in situ optical sensing devices.

Parameter	Wavelength	Proxy For	Calibrant	Ref.
*Fluorescence Spectroscopy*				
Tryptophan-like fluorescence (TLF)	λ_ex_ = 280 nm λ_em_ = 365 nm	biological activity, microbial contamination with	L-Tryptophan	[21]
Humic-like fluorescence (HLF)	λ_ex_ = 280 nm λ_em_ = 450 nm	autochthonous (within stream algal and microbial activity) and allochthonous (soil-derived organic matter) generation of small colloidal and dissolved organic matter	Quinine sulfate	[22]
Fluorescent DOM (FDOM)	λ_ex_ = 325 nm λ_em_ = 470 nm	total DOC concentration	Quinine sulfate	[23]
Chlorophyll a (f-Chl a)	λ_ex_ = 430 (470) nm λ_em_ = 675–750 nm	biomass of algae	Dyes, pure or extracted Chlorophyll a	[24]
Phycocyanin (f-PC)	λ_ex_ = 590 nm λ_em_ = 640–690 nm	biomass of cyanobacteria	Phycocyanin	[25]
Fluorescence index (FI)	λ_ex_ = 370 nm λ_em_ = 470 and 520 nm	microbial (high FI~1.8) or terrestrial (low FI~1.2) source of DOM	$FI=\frac{Intensity\;\lambda_{em\left(470\right)}}{Intensity\;\lambda_{em\left(520\right)}}$	[26]
*Scattered light measurement*				
Turbidity	portion of light scattered at angle 90° from the incident beam (λ > 800 nm)	loss of clarity in water	Formazin turbidity standard	[27]
*UV/Vis spectroscopy*				
Nitrate	A_217–240 nm_	eutrophication of freshwater ecosystems	NO_3_-N	[7]
Spectral absorption coefficient (SAC_254_)	A_254 nm_	organic loads of water		[9,28]
Colored dissolved organic matter (CDOM)	A_254 nm_ or A_370 nm_	colored and photoactive fraction of DOM	$TOC=0.492\;A_{250}$ $-1.23\;A_{364}+1.83$	[29,30]
Chemical oxygen demand (COD)	A_225–260 nm_	pollution of water by reducing substances’		[31]
Phycocyanin (PC)	A_615 nm_ and A_652 nm_	cyanobacterial components	$PC=A_{615}-0.474\frac{A_{652}}{5.34}$	[32]
Turbidity	A_>800 nm_	loss of clarity in water	Formazin turbidity standard	[27]

A: absorption coefficient, λ_ex_: excitation wavelength, λ_em_: emission wavelength.

In addition to developments in laboratories, a number of commercially available sensor systems based on optical spectroscopy can be used for different analytical purposes. A summary of the common online UV/Vis instruments for water quality monitoring is shown in [33], while Lee et al. also summarize UV/Vis and DOM sensors [23]. Sensors for nitrate, algae, tryptophan or chlorophyll are available from different manufacturers. 

However, the majority of these sensor probes are characterized by a predefined configuration, a fixed hardware setup and static data processing methods. Therefore, new applications developed in the laboratory cannot simply be realized with the existing sensors in the field.

For this reason, we developed a compact field-deployable optical instrument using fluorescence, absorbance and scattering to identify and quantify contaminants and natural substances in water bodies. The aim of this development was to simultaneously ascertain as many of the parameters listed in Table 1 as achievable. Due to the application of a broadband light source in UV/Vis spectroscopy and detection with a miniature spectrometer with a wavelength range between 225 and 1000 nm, all the parameters listed in Table 1 for UV/Vis spectroscopy can be detected by taking one measurement. Our LED module with four slots for different LEDs permits excitation with four wavelengths, while the emission spectra can be observed over the whole wavelength range, in contrast to the use of a simple photodiode. As the measurements generate data at a high velocity and in a high volume, new concepts in data processing and data transfer were realized. The sensor platform developed is characterized by the following features:

- Synchronous data acquisition: UV/Vis and fluorescence measurements can be an-alyzed in one step. The absorbance measurement is made in a 180° configuration while fluorescence emission is measured in 90° geometry. The path length of the measurement cell is 10 mm. The water sample is pumped through the measuring cell. Due to its position inside the sensor probe, external interfering influences were minimized. The spectrometer permits detection over the entire wavelength range.
- Adaptable hardware configuration: To adapt the sensor probe to different aquatic conditions, the sensor configuration can be easily changed. This includes the replacement of light sources and the adjustment of their intensities. The operational conditions of the spectrometer and the integration parameters can also be easily changed.
- Open data processing platform: The integrated processing platform facilitates the further handling and fusion of the spectral data (quantification, turbidity compensation, qualitative and quantitative assessment of water quality information). All data are available and adjustable for users at each level of processing.
- Open Data Model: Processed data, measurement methods and metainformation are stored in a holistic structure. All these data can be transferred by the user.
- Data visualization: The data are displayed in real time on a dashboard for analysis and pattern identification.
- Remote control: A specially programmed app enables access to the sensor probe. It allows the monitoring of operating status, the definition of measurement intervals and times, as well as the execution of functional tests on the light sources. Furthermore, the app shows quantitative results of predefined analytes.

Our developed sensor probe was tested in the lab by calibrating it with the substances mentioned in Table 1. Additionally, a field test was carried out for verifying the long-term stability.

## 2. Materials and Methods

### 2.1. Design and Development of the UV/Vis–Fluorescence Submersible Sensor Probe

#### 2.1.1. Hardware Development

**Figure 1:**
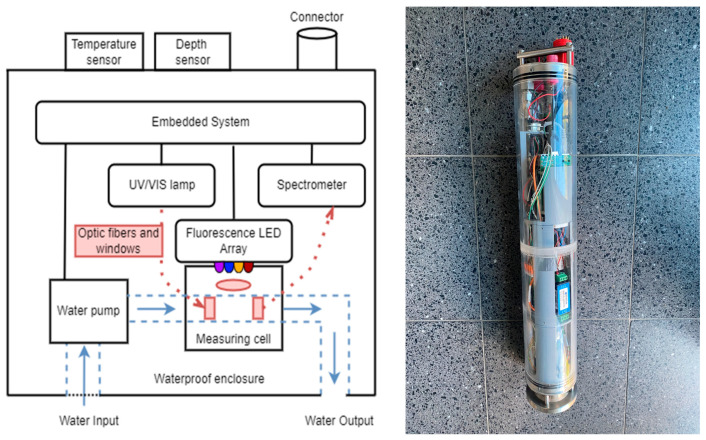
Sensor concept and final UV/Vis–fluorescence submersible sensor probe.

The UV/Vis–fluorescence submersible sensor probe is integrated into a watertight enclosure with a cylindrical design, which is made of acrylic glass. With a height of 590 mm and an outer diameter of 100 mm, the sensor probe can be used for measurements in water bodies and groundwater wells. The general operational principles are shown in Figure 1. 

**Figure 2:**
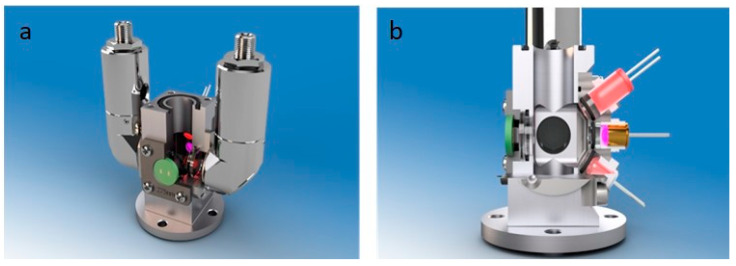
Measuring cell with two 90° collimators (**a**) and LED array (**b**).

The measuring cell with a pathlength of 10 mm is located inside the submersible sensor probe. The water sample is pumped through the measuring cell and then expelled through the water outlet using a self-priming brushless micropump (MGD1000F, TCS Micropumps, Faversham, UK). A filter with a mesh size of 1 mm is integrated into the inlet in order to protect the sensor system from particles. The pump can be stopped during the measurements. This function is integrated into the automated measurement process. A miniaturized deuterium–tungsten light source FiberLight D2 (Heraeus, Hanau, Germany) is used for the measurement of transmittance in the UV/Vis range. The continuous spectra of deuterium (UV) and tungsten (Vis) are guided using an optical fiber to a collimator. Short solarization-resistant FG600AEA optical fibers (Thorlabs, Newton, NJ, USA) with a diameter of 600 µm were used, which reduce UV absorbance and aging. Due to the limited space available, a 90° PL-25-12-90SS-SLIM2-CO collimator (Plasus, Mering, Germany) is used for the optical connection to the measuring cell (see Figure 2a). The measuring cell is sealed to the collimator using a 3 mm thick fused silica disk (Suprasil 2 Grade A, Aachener Quarzglas Technologie Heinrich, Aachen, Germany).

For the fluorescence spectroscopy, a self-developed LED array circuit is used as the light source. This circuit is mounted directly onto the measuring cell, offset 90° to the UV/Vis light source. The measuring cell has slots for four LEDs (see Figure 2b). Fused silica disks with a thickness of 3 mm were also used to seal the measuring cell to the LEDs.

The UV/Vis and fluorescence spectra were taken using a Qmini AFBR-S20M2WU spectrometer (Broadcom, San José, CA, USA) with a wavelength range from 225 to 1000 nm and a spectral resolution of 1.5 nm. The spectrometer is connected to the measuring cell via a fused silica disk and a 90° collimator (see Figure 2a). Both collimators are positioned opposite each other in a 180° geometry. 

The choice of a 180° configuration for the absorbance measurements and a 90° geometry for the fluorescence emission measurements resulted from the commonly used setups described in the literature [16]. UV/Vis or transmission measurements are always carried out in a 180° configuration [33]. For fluorescence detection, additional optical elements such as mirrors should be avoided in the design, and the greatest possible sensitivity should be achieved. The 90° angle between the excitation and detection is realized in almost all laboratory devices, without which our rectangular measuring cell is not feasible.

The UV/Vis and four fluorescence spectra are recorded one after the other after the pump is stopped. The entire measurement process takes less than 30 s.

All the electronic components in the submersible sensor probe are controlled by a Raspberry Pi 4 Model B (Raspberry Pi Foundation, Cambridge, UK). The spectrometer is connected with the embedded system via the USB interface. Communication with the spectrometer is facilitated using the manufacturer’s provided software development kit. Additionally, a custom-designed add-on board enables the seamless integration of all hardware components into the embedded system. Notably, the digital interfaces for the sensors are positioned along the outer edge of the add-on board for convenient accessibility and efficient operation. 

On the top of the enclosure, a TSYS01 high-accuracy, fast-response temperature sensor (Blue Robotics, Torrance, CA, USA) and a Bar30 pressure sensor (Blue Robotics, Torrance, CA, USA) are integrated. These sensors can be used to measure the water temperature and the depth of the UV/Vis–fluorescence sensor probe. Both sensors are connected via the I2C interface to the add-on board. The sensor probe receives a reliable 12 VDC power supply and establishes an Ethernet connection via the DBH13MSS connection terminal (MacArtney, Esbjerg, Denmark). To oversee the LED array within the measuring cell, the add-on board incorporates a TLC59108F driver from Texas Instruments (Dallas, TX, USA). This integrated driver offers the capability to individually parameterize each LED’s current, allowing for precise adjustment of brightness across 256 gradations.

**Figure 3:**
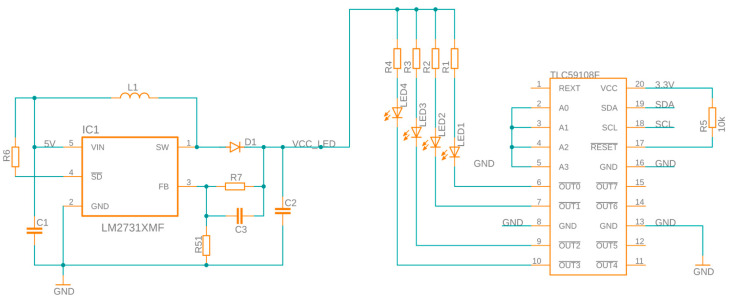
Schematic of the LED driver.

To achieve higher intensities using a higher current, the LED driver can also pulse the current. Since not every LED can be supplied with the same voltage, a separate power supply circuit is integrated into the add-on board. The LM2731 (Texas Instruments, Dallas, TX, USA) hardware component is used for this purpose and act as a boost converter. The maximum current is separately adjustable using a series of resistances for each LED. The developed LED driver is schematically shown in Figure 3.

#### 2.1.2. Software Development

**Figure 4:**
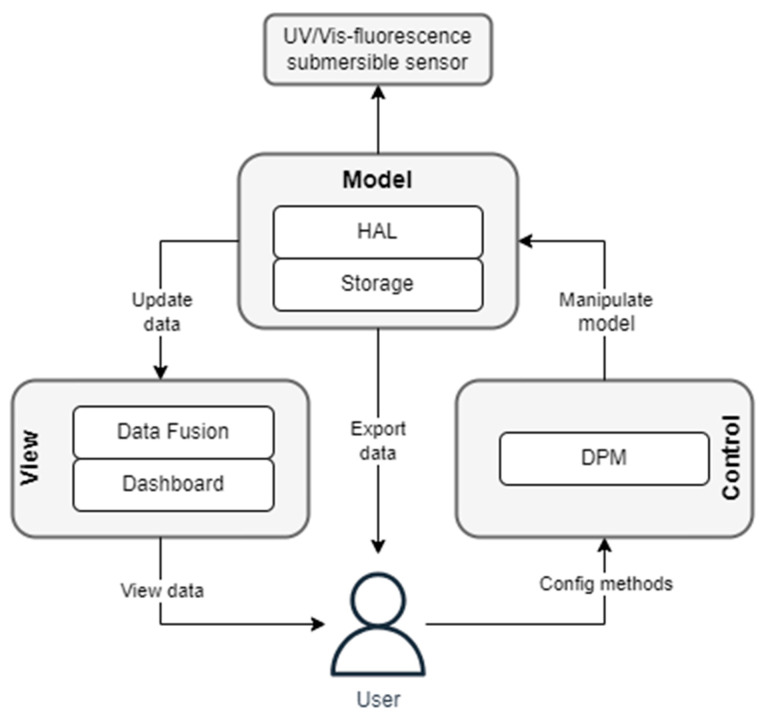
Flow diagram of Model–View–Controller architecture.

To handle the hardware configurations, the UV/Vis–fluorescence submersible sensor probe is equipped with a flexible data processing framework. This includes four steps: data collection, data processing, data storage and data visualization. These tools are integrated into an embedded system. To enable a flexible data processing framework, a Model–View–Controller architecture is used for the software development. This approach separates the software architecture into the three parts: Model, View and Controller (Figure 4). The data management is provided by the Model module. This module consists of two components, the Hardware Abstraction Layer (HAL) and the data storage.

The HAL is designed for connecting all sensors and actuators using a standardized interface for communication with all components within the UV/Vis–fluorescence sensor probe. The HAL has a web interface based on the HTTP protocol allowing flexible access to the hardware. HTTP request parameters can be used for the adaptation of the sensor and actor settings. Based on the hardware configuration, it is possible to adjust the integration time of the spectrometer, the averaging of the spectra by a user-defined number of iterations and control of the light sources with different intensities. The second component is the data storage model. The data storage model combines all spectral data. To change the settings or to manipulate the model, the user can modify the process via the Controller module. The Controller module is specified by the data processing method (DPM). The DPM provides a software framework for the integration of the methods, which have been developed in the laboratory. The software Node-RED (OpenJS Foundation, San Francisco, CA, USA, Version 3.0.2) is used for this task. 

Due to the flow-based programming framework, laboratory methods can be integrated in a flexible way. DPM based on Node-RED offers also the advantage that a large library of data processing tools can be created and used. If the UV/Vis–fluorescence sensor probe is ready for use, the user can post-process (Data Fusion) and display (Dashboard) the results via the View component. With data fusion, indicators can be calculated within a spectrum but also between different spectra. This makes it possible to determine the fluorescence index (FI), as well as other indicators published in the literature. These post-processing steps can be carried out directly using the Grafana software (Version 10.1.5). In addition, self-developed scripts can also be stored and executed in the View component. This allows an individual evaluation of spectra according to self-defined parameters. A summary of the used software components is shown in Table 2.

#### 2.1.3. Field Application Setup

The successful use of the sensor probe in the field requires some additional modifications to ensure seamless functionality and reliable acquisition and transmission of data. This includes the integration of a power supply and a data communication unit.

**Figure 5:**
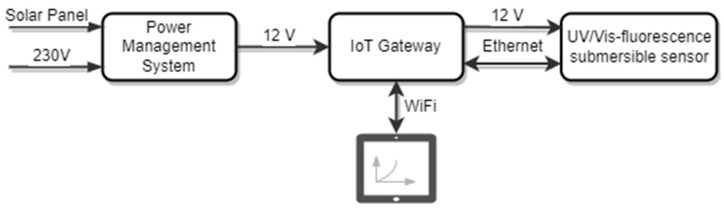
Schematic structure of sensor configuration for field applications.

For this purpose, a field-deployable data and power management unit was de-signed. As it is equipped with a BlueSolar MPPT 100 solar charge controller (Victron energy, Almere, The Netherlands), an industrial NPC24-12i battery (Yuasa, Krefeld, Germany) and an MPM-90-12ST AC–DC power supply unit (MEAN WELL, New Taipei City, Taiwan), the system can be operated energy self-sufficiently as well as with conventional main voltage. The integrated RUT955 IoT gateway (Teltonika, Kaunas, Lithuania) establishes the Ethernet connection to the sensor probe and provides a Wi-Fi access point for accessing the data processing platform. When connected to this network, the user can interact with the control and display module via a web interface. A schematic structure of the field configuration is illustrated in Figure 5. The modular design of the sensor probe allows its use in different scenarios, with stationary and mobile profile measurements. Additionally, a specially designed Grafana dashboard visualizes all the water parameters recorded by the sensor probe.

### 2.2. Lab Validation

#### 2.2.1. Preparation of Water Samples

As summarized in Table 1, different substances and sum parameters are routinely measured using UV/Vis and fluorescence sensor probes in aquatic systems. The determination of the sum parameters (e.g., DOM) requires calibration with suitable single compounds. Our developed sensor probe must be able to detect these substances with sensitivities as described in the literature or as they correspond to the specifications of commercial devices. For this purpose, we prepared standard solutions in water. Stock solutions of formazin turbidity standard (4000 NTU, Hach, Manchester, UK), quinine sulfate (Sigma-Aldrich, St. Louis, MO, USA), L-tryptophane (Sigma-Aldrich, St. Louis, MO, USA), humic acid (Carl Roth GmbH, Karlsruhe, Germany) and potassium nitrate (Merck, Darmstadt, Germany) were diluted, so that a series of standard solutions within a range of below 1 mg L^−1^ to 12 mg L^−1^ (100 mg L^−1^ in case of nitrate) was obtained. 

#### 2.2.2. LED Array Configuration

Different target analytes in fluorescence spectroscopy require different excitation wavelengths and therefore different LEDs. As described, the developed LED array can be equipped with four different light sources of different wavelengths. These can be changed easily in order to modify the sensor to changing measuring tasks. The current supplied for each LED can be adjusted in accordance with their maximum power dissipation for an optimal performance. Based on these maximum power dissipations, the current of each LED was pulsed or set constant. It is important to note that the LED driver has a maximum current capacity of 120 mA. This limit is taken into consideration when configuring the current settings for the LEDs. Table 3 summarizes the LEDs and their specifications which were used for the validation experiments. We used industrial-standard LEDs with a uniform housing.

#### 2.2.3. Data Collection and Data Processing

The flow cell was adapted for the lab validation experiments. Before measuring the standard solutions, blank spectra were taken with pure water. These blank spectra are stored on the embedded system and are available for any further preprocessing steps. Sequentially, the prepared standard solutions were filled into the flow cell. The measurement process begins with UV/Vis spectroscopy and the activation of the deuterium–tungsten light source. After an interval of one second, 10 spectra were acquired and then averaged. The spectrometer operates with an integration time of 160 milliseconds for the UV/Vis measurements. After the measurement, the light source is deactivated and the fluorescence measurement starts. Depending on the target analyte, the corresponding LED from Table 3 is used. While the LED light source is active, the spectrometer records five corresponding spectra and an average value is computed from these five spectra. Here, the integration time is four seconds. Three series of measurements were carried out for each substance and for each spectroscopy method. The data collection and data processing method are implemented into the Control module. Therefore, Node-RED flows have been developed. The results are saved by the Model module and made available for export via the View module. The Node-RED HTTP modules are used to address the UV/Vis–fluorescence submersible sensor via the HAL. The integration time and the number of measurements per spectrum are set using the HTTP parameters. A measured spectrum is converted into a key-value pair so that it can be processed as a JavaScript object. Supplemented with meta information (time, location, water temperature, depth), the sample site is written into the database via the Node-RED Influx module. The light sources can also be controlled via the HTTP module. 

## 3. Results

### 3.1. Lab Validation

**Figure 6:**
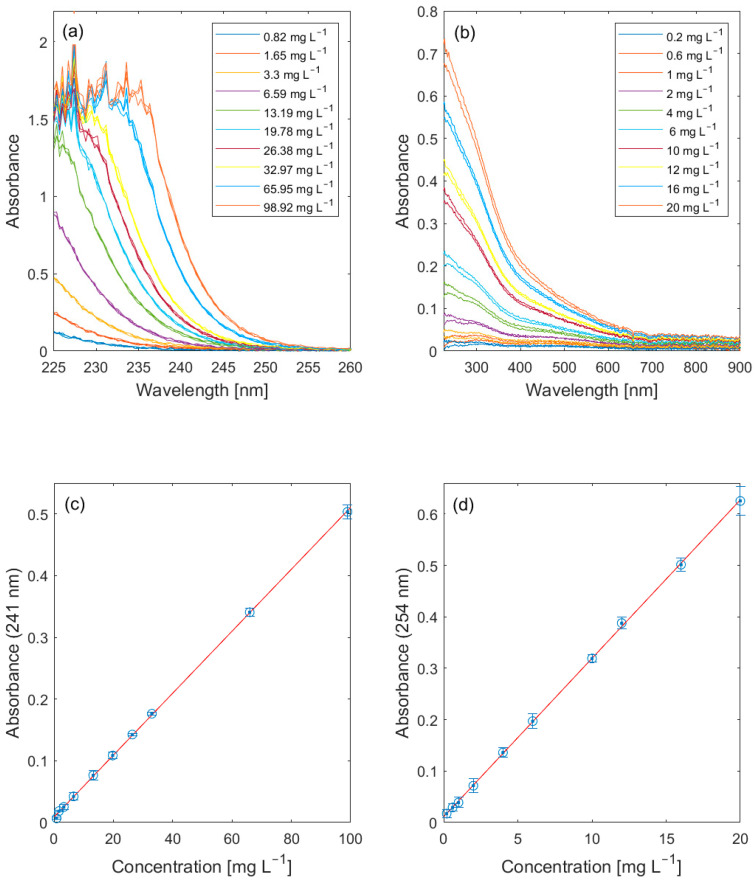
Spectra of (**a**) nitrate and (**b**) humic acids and results of quantitative measurements of (**c**) nitrate (peak intensities at 241 nm, concentrations as NO_3_-N) and (**d**) humic acids (peak intensities at 254 nm) using UV/Vis spectroscopy.

**Figure 7:**
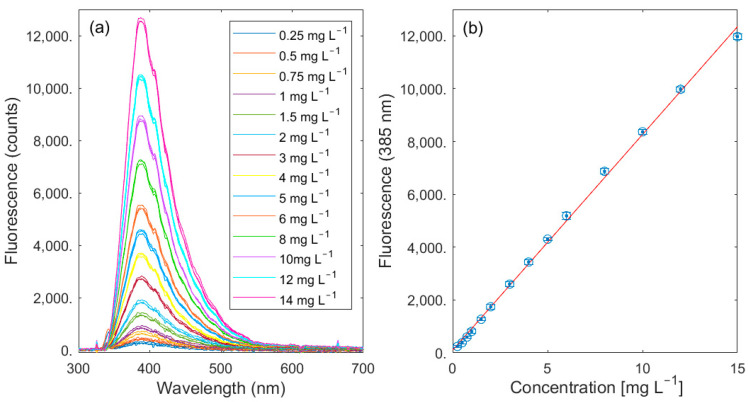
Fluorescence emission spectra of quinine sulfate (**a**) as proxy for FDOM after excitation at 340 nm and calibration (**b**) using peak intensities at λ_em_ = 385 nm.

**Figure 8:**
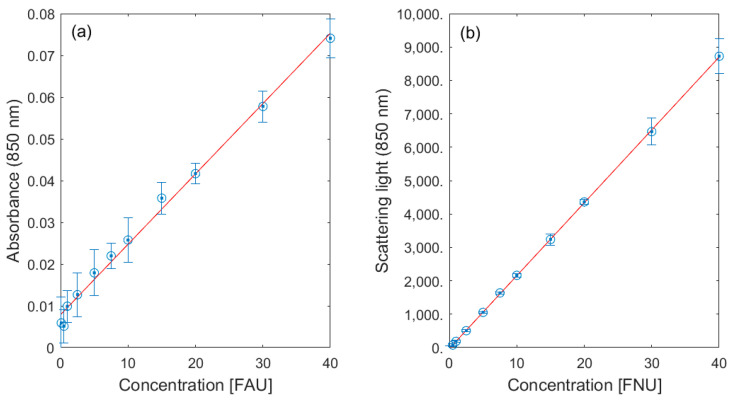
Results of turbidity measurements using UV/Vis spectroscopy (**a**) and using scattered light measurement (**b**).

All substances investigated can be clearly detected with intensities comparable to those of other lab devices or commercial sensor probes. Figure 6, Figure 7 and Figure 8 show the same examples for both UV/Vis and fluorescence spectroscopy. Figure 6 summarizes the quantitative absorbance measurements of nitrate and humic substances. 

Both target analytes are among the most frequently monitored parameters in aquatic ecosystems. Humic substances must be monitored, particularly in the catchment area of drinking water reservoirs. Nitrate pollutions belong to the most challenging and costly environmental problems due to the permanent release of nitrates into natural waters. High concentrations of nitrate can promote the growth of algae and phytoplankton. With limits of detection (LODs) of 1 mg L^−1^ for nitrate (Calculated as NO_3_-N) and 0.2 mg L^−1^ for humic acids, our sensor probe permits the sensitive detection of these parameters. The LODs were calculated according to [34]. As can be seen from the spectra, high concentrations of humic acids complicate the evaluation of nitrate concentration. However, this is a general limitation of the method (UV/Vis spectroscopy) and not a special feature of our sensor probe. The advantage of our sensor probe is that possible interferences can be seen from the complete spectra, while simple sensor probes with photodiodes as detectors let these influences go unnoticed.

FDOM is another essential parameter for characterizing natural waters and serves as a significant proxy for the total DOC concentration. As stated in Table 1, the calibration is conducted using quinine sulfate and the FDOM concentrations can be given as quinine sulfate units (QSUs) where 1 QSU = 1 ppb quinine sulfate. The results of our measurements using fluorescence spectroscopy are summarized in Figure 7. The values for excitation wavelength were taken from the literature [23]. The fluorescence emission at 385 nm was used for calculating the calibration line. A LOD of 0.3 mg L^−1^ was calculated for quinine sulfate. 

According to ISO 7027-1:2016, turbidity is another important parameter for the assessment of water quality. As stated above, turbidity can be measured in transmittance (850 nm) or using scattered light measurement (λ_ex_ = λ_em_ = 850 nm) depending on the corresponding concentration range. As expected, scattered light measurements provide a more sensitive detection with a LOD of 0.2 FNU while transmittance measurement has a LOD of 2.4 FAU. Although our probe does not achieve the sensitivity of laboratory devices with LODs (<0.05 FNU), these LODs are well suited to sensitive turbidity measurement in the field. 

### 3.2. Results from Field Tests

In addition to laboratory validation, the sensor probe was applied in the field as part of a water monitoring campaign along the Elbe River. During this campaign, the primary focus was on the measurement of two key parameters: turbidity and FDOM. 

**Figure 9:**
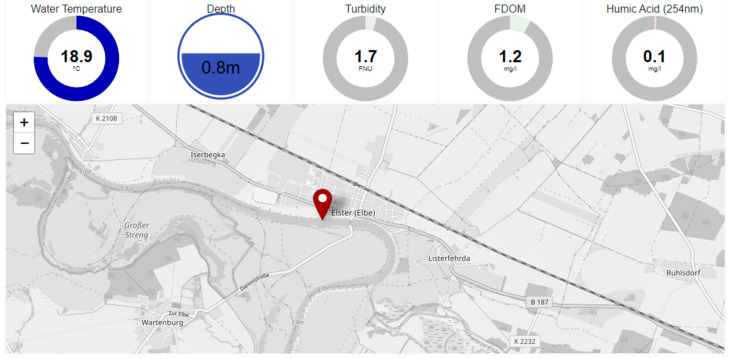
Dashboard for water monitoring campaign on the Elbe River. Water temperature, water depth, turbidity and FDOM are displayed together with the geo-position.

For this purpose, our sensor probe was equipped with the corresponding LEDs. The developed dashboard (Figure 9) permits it use as a pivotal tool for real-time data visualization and management during the campaign. Besides displaying the fluorescence measurement values, additional data on water temperature and water depth are integrated. Furthermore, the dashboard shows a map section where the current position of the measurement is displayed. 

**Figure 10:**
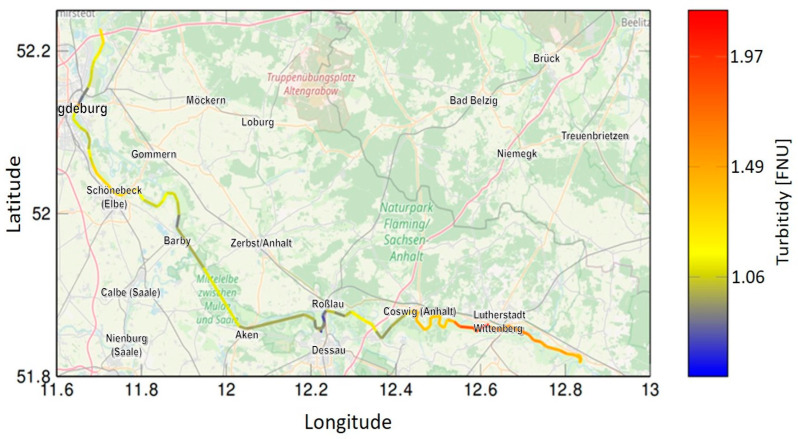
Spatial data plot of the turbidity measurements of the monitoring campaign at the river Elbe (315 single measurements).

The user receives all the essential information and results in real time via this dashboard. It is also possible to download the results via data export for further post-processing procedures. A result of data processing can be seen in Figure 10, where geographical coordinates (longitude and latitude) have been combined with turbidity data to create a spatial data plot. Such procedures can be used to visualize concentration profiles, as shown in the example, and allow the user to quickly identify anomalies and significant changes in concentration. 

## 4. Conclusions

The focus of this work was the development of an integrated sensor platform for UV/Vis and fluorescence spectroscopy which can be used for analyzing a wide range of unsaturated and aromatic compounds in aquatic ecosystems. The sensor platform is defined by the six requirements mentioned in the introduction. For simultaneous use of UV/Vis and fluorescence spectroscopy, a new measurement cell was designed, which is located inside the sensor probe to minimize external influences on the water sample. Four LEDs and a broadband light sources are attached to the measurement cell. The LEDs can be easily changed by the user for a high degree of flexibility in the configuration. The developed adjustable LED driver allows the use of various standard LEDs with the desired wavelength. An open processing framework is based on this hardware. The Model–View–Controller architecture allows for the clear separation of key features, easy modifications and independent advancements of the modules. With the capability for direct data fusion and data presentation in the View module, the measurement results can be presented in a clear and concise manner within field applications.

## Figures and Tables

**Table 2:** Software stack for the Model–View–Controller architecture.

Module	Component	Software
View	Data Fusion Dashboard	Grafana
Model	HAL Storage	Python-FastAPI InfluxDB 2.0
Control	DPM	Node-Red

**Table 3:** LED array configuration.

LED	Excitation Wavelength [nm]	Current [mA]	Parameter
DUV-HL5N, Roithner LaserTechnik GmbH	340	40 (pulsed)	DOM
VL440-5-15	440	100 (pulsed)	Chlorophyll a
CY5111A-WY, Roithner	590	100 (pulsed)	Phycocyanin
OP265FAB, TT Electronics	850	120 (constant)	Turbidity

## Data Availability

The analytical results and data are available from the corresponding author upon request.
